# Adaptive Fine Distortion Correction Method for Stereo Images of Skin Acquired with a Mobile Phone

**DOI:** 10.3390/s20164492

**Published:** 2020-08-11

**Authors:** Cho-I Moon, Onseok Lee

**Affiliations:** 1 Department of Computer Science & Engineering, Graduate School, Soonchunhyang University, 22, Soonchunhyang-ro, Asan City, Chungnam-do 31538, Korea; chdl813@sch.ac.kr; 2 Department of Medical IT Engineering, College of Medical Sciences, Soonchunhyang University, 22, Soonchunhyang-ro, Asan City, Chungnam-do 31538, Korea

**Keywords:** pincushion distortion, distortion correction matrix, the mobile stereo image, the mobile skin image

## Abstract

With the development of the mobile phone, we can acquire high-resolution images of the skin to observe its detailed features using a mobile camera. We acquire stereo images using a mobile camera to enable a three-dimensional (3D) analysis of the skin surface. However, geometric changes in the observed skin structure caused by the lens distortion of the mobile phone result in a low accuracy of the 3D information extracted through stereo matching. Therefore, our study proposes a Distortion Correction Matrix (DCM) to correct the fine distortion of close-up mobile images, pixel by pixel. We verified the correction performance by analyzing the results of correspondence point matching in the stereo image corrected using the DCM. We also confirmed the correction results of the image taken at the five different working distances and derived a linear regression model for the relationship between the angle of the image and the distortion ratio. The proposed DCM considers the distortion degree, which appears to be different in the left and right regions of the image. Finally, we performed a fine distortion correction, which is difficult to check with the naked eye. The results of this study can enable the accurate and precise 3D analysis of the skin surface using corrected mobile images.

## 1. Introduction

The skin surface is composed of a complex network structure made up of wrinkles. We can analyze the skin conditions by observing the morphological (wrinkle width, skin plateaus area, etc.) and topology changes (wrinkle depth, etc.) in the structure of the skin surface. Three-dimensional (3D) analyses of skin conditions, such as roughness and texture, are required to accurately diagnose skin aging and skin diseases [1,2,3,4,5]. Stereophotogrammetry is a 3D analysis technique used to evaluate the topology and morphology of the skin surface. It is an objective, non-invasive technique that is used to identify skin surface features from two different perspectives and estimate depths through triangulation. This technique can estimate depth values of less than a millimeter and can thus be effectively utilized for the 3D analysis of the skin surface structure [6,7,8]. In this study, we established a non-convergence model using a mobile camera and acquired stereo skin images for a 3D skin analysis. Then, we extracted the three-dimensional information and disparity value through stereo matching and confirmed the depth resolution in the micro range of the mobile image.

In addition to being cost-effective and highly usable and having excellent hardware, mobile phones are capable of obtaining high-resolution images anywhere and at any time, thus being useful in various fields of study, particularly biometrics [9,10]. Through our prior study [11], we confirmed a high-performance mobile camera can acquire a high-resolution close-up image that can be observed at up to a micrometer resolution.

Most current mobile phones are being manufactured with wide-angle cameras that can capture wide range images in addition to high-quality and high-resolution images. The use of wide-angle cameras in mobiles has led to strong distortions in mobile images [12]. Lens distortion results in changes in the curvature or geometry of an image. It strongly occurs toward the boundary of the image. Different types of lens distortions include radial, decentering, and thin prism distortions. Radial distortion is the most common type of distortion and appears in the form of a barrel or pincushion distortion [13].

Many studies have attempted to correct non-linear geometric image structures caused by barrel distortion in the images from wide-angle cameras. One study obtained camera parameters by the calibration of the camera to calculate the lens distortion, which was then used to correct the distortion in the image [14]. Other studies proposed a distortion correction method using image information, such as lines, without a calibration pattern [15,16]. Recently, many studies have been conducted to correct distortion by using polynomial and division models as distortion correction methods that are not based on camera parameters. Additionally, other distortion correction studies have applied the piecewise polynomial function and circle fitting method to improve the performance of the lens correction model [13,17]. However, pincushion distortion does not significantly affect the image. Therefore, while most studies deal with barrel distortion correction in wide-view images, pincushion distortion is rarely studied.

In this study, we acquire mobile images affected by pincushion distortion of the proximity area, generate a distortion correction matrix (DCM) using the division model, and propose a correction method by pixel for the close-up mobile image. We then evaluate the performance of the proposed correction method by conducting a matching analysis of corrected mobile stereo images. The fine geometric distortions appearing on the mobile image of the skin surface are then corrected. The study’s contribution is as follows.

1. We propose the distortion correction method for mobile images as a pre-processing step for the 3D evaluation and analysis of stereo skin surface images captured using a mobile phone.
2. Unlike prior studies that correct the mobile image of the human face, environment, etc., we correct the distortion of skin surface images with a complex structure to demonstrate the usability of mobile cameras in the field of biometric signals, which requires precision at the µm level.
3. Most studies deal with the correction for barrel distortion, which appears strongly over a wide area, such as in fish-eye lens images. Contrastingly, our study proposes a correcting method for fine pincushion distortion in close-up images.
4. We employ the division model using a single parameter rather than the camera calibration approach, which is a complicated and time-consuming method, to correct the mobile image in real time. Additionally, we generate a pixel-by-pixel distortion correction matrix containing different degrees of distortion in the left and right regions of the image to increase the precision of distortion correction.

## 2. Materials and Methods

### 2.1. Image Acquisition

We use a Galaxy A3 (SM-A310NZKAKOO, Samsung Electronics Co., Suwon. Korea). Its rear camera has the specifications of 1.3 MP, aperture F1.9. It has a low-cost, high-performance camera. We zoomed in on the camera to acquire a narrow range, like the region on the back of the hand, without additional equipment.

In this study, we acquired three types of mobile image with a non-convergence stereo system using the mobile phone: the vertical line pattern image in plane, the standard scalar bar image including depth, and a skin surface image.

We took a zoomed-in 2.0 times image with an aspect ratio of 4:3 from a working distance of 60 mm to 80 mm between the camera and the ground. We acquired a total of 5 sets of stereo images, where the baseline—the distance between the stereo images—is 10 mm, by adjusting the working distance by 5 mm intervals. Stereo images consist of the reference image and the shift image moved by 10 mm from the baseline. For the analysis of the skin surface on the back of the hand, it is effective to obtain the center of the image, which has a small effect of the skin curvature. Thus, the baseline value of 10 mm is an adequate distance that includes a flat skin surface area.

We adjust the geometric changes of the mobile image. Thus, for pre-processing we would not consider image information such as color other than pixel position. Therefore, our study uses mobile images converted to gray-scale using MATLAB^®^ (R2018b, The Mathworks, Cambridge, UK).

### 2.2. The Vertical Line Pattern

**Figure 1:**
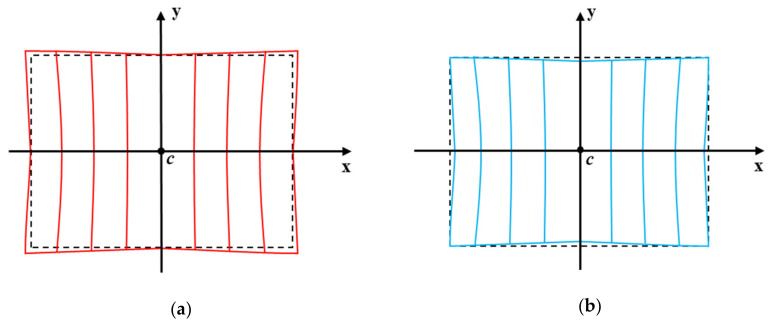
Type of pincushion distortion. (**a**) Negative pincushion distortion, (**b**) positive pincushion distortion.

A pincushion distortion can be of two types—positive and negative (Figure 1). Most studies have focused on negative displacement pincushion distortion, where distortion occurs outside the observable range [18]. The correction of negative pincushion distortion may lead to a loss of information in the image. However, the close-up mobile image in this study presents a positive displacement pincushion distortion, which occurs inside the observable range. The correction of this image generates a hole region on the image, where no image information is present. Because a hole in the image degrades the image quality, it is important to interpolate a hole region as similar as possible to the raw image. We calculate the distortion parameter based on the assumption that the principal point—i.e., the optical axis of the intersection in the image plane—is the center of the distorted image [19]. We acquire mobile stereo images that have the same epipolar line and find the corresponding point located on the horizontal axis of the left and right images. Therefore, a vertical line pattern matrix with 1 mm intervals is created to investigate the position transition of each corresponding point using the MATLAB software. The ideal vertical line pattern image utilizes the self-produced matrix information that had an identical resolution, taking into account the real mobile image. The distorted vertical line pattern image is obtained by taking a picture of this pattern. After rearranging the vertical lines based on the center of the two images (c point in Figure 1), we calculate the distortion of the mobile image by comparing the distorted and undistorted (ideal) vertical line groups.

### 2.3. Proposing the Distortion Correction Matrix (DCM)

In this study, we perform distortion correction using the division model proposed by Fitzgibbon [20], considering an ideal point ($x_{u}$, $y_{u}$) and a distorted point ($x_{d}$, $y_{d}$). Traditional distortion models use the division distortion coefficients $\lambda_{1}$ and $\lambda_{2}$. Equation (1) represents traditional distortion models. $r_{d}=\sqrt{{\left(x_{d}-x_{c}\right)}^{2}+{\left(y_{d}-y_{c}\right)}^{2}}$ denotes the Euclidean distance between the distorted point and the center point ($x_{c}$, $y_{c}$) of the image, where $x_{c}=image\;width/2$ and $y_{c}=image\;height/2$. Additionally, $r_{u}=\sqrt{{\left(x_{u}-x_{c}\right)}^{2}+{\left(y_{u}-y_{c}\right)}^{2}}$ denotes the Euclidean distance between the ideal point and the image center.

(1) $$\left(\begin{array}{c} x_{u}\\ y_{u} \end{array}\right)=\left(\begin{array}{c} x_{d}\\ y_{d} \end{array}\right)/\left(1+r_{d}^{2}\lambda_{1}+r_{d}^{4}\lambda_{2}\right).$$

We generate the distortion correction matrix (DCM) by calculating the relationship between the ideal and distorted coordinates using the division model and distortion parameter. We then perform forward mapping from the distorted point ($x_{d}$, $y_{d}$) to the ideal point ($x_{u}$, $y_{u}$) using the DCM.

First, we calculate the distortion ratio (R) using the distorted and ideal points. The value of R is obtained by calculating the degree of distortion of each pixel using $r_{u}$ and $r_{d}$. As the acquired mobile image presents a pincushion distortion, R>0. Additionally, the larger the distortion, the larger the value of R. We then calculate a distortion coefficient (k) reflecting the distortion ratio.

(2) $$R=\frac{r_{u}}{r_{d}},\;k=\frac{1-R}{r_{d}^{2}}.$$

The traditional division model equation consists of 4th order terms, but backward mapping can ignore error terms higher than the 4th term and represent an approximate value through the 2nd order term of $r_{d}$ [21,22,23]. Accordingly, we apply the distortion coefficient (k), calculated by using the squared term of $r_{d}$ and the distortion ratio (R), to the forward mapping. Thus, we utilize the division model equation, which is reduced to a second-order polynomial using the value of k. The distortion correction coefficient (λ) is calculated for each pixel using the following equation.

(3) $$\lambda =\frac{-1+\sqrt{1+4kr_{u}^{2}}}{2kr_{u}^{2}}.$$

Finally, the distorted point is relocated to a corrected point ($x_{n}$, $y_{n}$) based on the image center ($x_{c}$, $y_{c}$). The new position of the corrected point denotes the resulting point approximated to the undistorted, ideal point.

(4) $$\left(\begin{array}{c} x_{n}\\ y_{n} \end{array}\right)=\lambda \left(\begin{array}{c} x_{d}\\ y_{d} \end{array}\right)+\left(\begin{array}{c} x_{c}\\ y_{c} \end{array}\right)\approx \left(\begin{array}{c} x_{u}\\ y_{u} \end{array}\right).$$

We obtain the DCM by calculating λ at each pixel in the distorted coordinates, thus correcting the distortion of mobile stereo images at different image angles using the DCM.

### 2.4. Interpolation of the Corrected Image

After correcting the negative pincushion distortion, a hole region appears in the image, which degrades the quality of the corrected image. We thus fill this region using the nearest neighbor interpolation method. The hole region occupies about 0.2% of the total pixels in the corrected image. The hole region is proportional to the degree of distortion. In other words, the distortion of the mobile image is very fine. As the region occupied by the hole appears to be very small, it is possible to obtain an interpolated image similar to the original image using the closest pixel value. Additionally, because the range of the hole is small, the pre-hole pixels and subsequent pixels are continuous pixels forming the skin surface structure elements, wrinkles and wrinkled cells. Therefore, we can obtain a natural image by interpolating the hole region using the neighboring pixels.

### 2.5. Regression Analysis of the Relationship between the Distortion Ratio and Different Angle Images

We calculated the degree of distortion in the mobile images captured at different angles of view at five different working distances. We can confirm the different angle of view of the images according to the working distances. We then performed a linear regression analysis on the relationship between the angle of view of the image and the distortion ratio obtained, using the results of the correspondence point matching in mobile stereo images via Minitab v19 (Minitab Inc., Coventry, UK).

## 3. Results

We evaluated the performance of the correction method by applying DCM to each acquired mobile image. First, for the vertical line pattern image in plane, we compared the before-and-after distortion correction and performed a linear regression analysis on the distortion differences in the image according to the angle of view. Next, after correcting the standard scalar bar image including depth, we confirmed the disparity, 3D information, which varies depending on the different heights, through stereo matching.

### 3.1. Comparing the before-and-after Distortion Correction for Vertical Line Pattern

**Figure 2:**
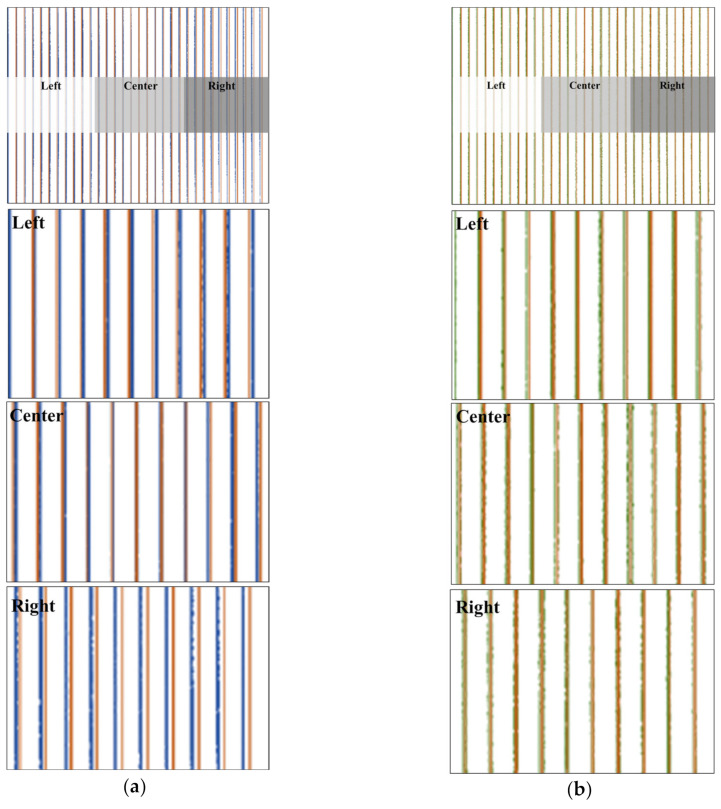
Comparison of the degree of distortion on the left, right, and central regions and before and after distortion correction. When compared with the ideal lines (in orange), the distorted lines (in blue) in (**a**) are corrected to the corrected lines (in green) in (**b**) to match the ideal lines. (**a**) overlap images of the ideal lines and distorted lines of the left, central, and right regions, (**b**) overlap images of the ideal lines and corrected lines of the left, central, and right regions.

Figure 2a shows the degree of distortion by comparing the undistorted ideal image and the original mobile image. Pincushion distortion was confirmed by the bend of the distorted line (in blue) toward the center of the image when matching the positions of the ideal undistorted lines (in orange) and the distorted lines. Additionally, we can observe the difference in the distortion ratio in the left and right regions relative to the center of the image. When observing the left, center, and right regions, as shown in Figure 2a, it is evident that the distortion in the right is stronger than that in the left. Figure 2b shows a comparison between the corrected lines (in green) and the ideal lines (in orange). Similarly, when observed from the left, center, and right regions, we can confirm that the corrected lines are relocated to match the ideal lines. Particularly, for the right region that seems to have a relatively stronger distortion, we can confirm that the gap between the blue lines and the orange lines decreases to near zero, which is the gap between the green lines and the orange lines. Through this, we confirmed that the correction distortion of the mobile image using DCM is performed well.

### 3.2. Comparing before-and-after Distortion Correction for Mobile Stereo Pattern Images in-Plane

Without camera lens distortion in acquired stereo images with a vertical line pattern in the plane, the corresponding lines in the left and right images always match, regardless of changes in the stereo baseline. However, when the image is affected by distortion, the vertical lines are geometrically deformed, and a gap appears between the corresponding lines of the left and right images.

**Figure 3:**
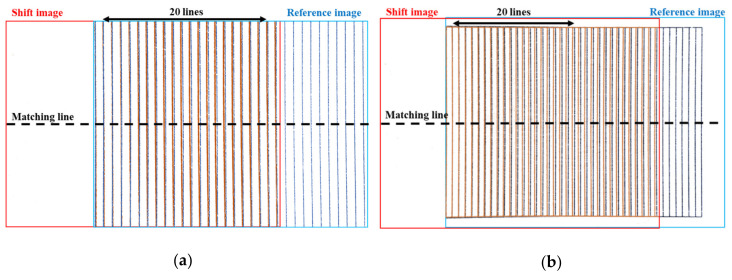
The combining image of the mobile stereo images in the plane (i.e., combining the reference image (in blue) and the shifting image (in red)). (**a**) Stereo images are acquired at a working distance of 60 mm and the baseline of 10 mm, (**b**) stereo images are acquired at working distance of 80 mm and the baseline of 10 mm.

In this study, we acquired a pair of stereo images which are captured from different angles of view at five different working distances. A pair of stereo images consist of the reference image and the shift image that is moved 10 mm laterally. Then, we compared the stereo images before and after the distortion correction by matching 20 corresponding lines in the shift and reference images. Figure 3a shows a pair of stereo images acquired at the shortest working distance of 60 mm for a stereo baseline of 10 mm, and Figure 3b shows a pair of stereo images acquired at the largest working distance of 80 mm for a stereo baseline of 10 mm.

**Figure 4:**
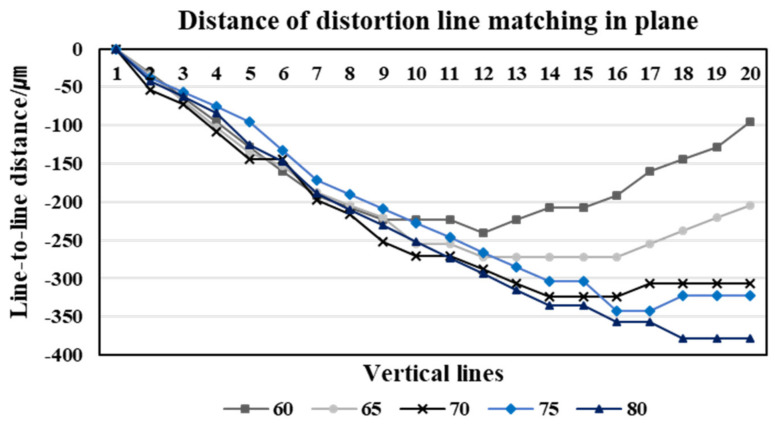
The stereo matching results of the distorted stereo image in a plane.

We matched the position of each corresponding line based on the first line at the matching line, which is the center line of the image, where the curvature of lines is the largest. If the image has no distortion, the position of the matching lines in the shift and reference images is the same—i.e., the distance = $x_{Li}-x_{Ri},\left(i=1,2,3,\ldots ,20\right)$ is zero. However, when looking at the graph of the stereo matching results of the position of the lines in a pair of distorted stereo images taken at the five different working distances (Figure 4), the distortion causes a gap between the corresponding lines in the stereo images. If the vertical lines are located at the boundary of the image, the effect of the distortion is stronger. Stereo matching of the two images is performed by comparing the position of the following line after matching the first line.

As we can see in Figure 3a,b, regardless of the different angles of view, the corresponding lines of the reference image are located in the left region of the image, highlighting that the distortion of the reference image is more affected. Therefore, the corresponding lines of the reference image are positioned toward the center of the image—i.e., curved towards the right. The distortion of the first corresponding line affects the subsequent matching lines, and thus the matching results show that $x_{Li}-x_{Ri}<0$ and that the distance value gradually increases toward the 20th corresponding line. From the reference image acquired at a 60 mm working distance, the distance between the corresponding lines increases gradually in the negative value. It then passes through the center region, where the distortion effect is rare, and then the distance decreases. For the image acquired at a working distance of 60 mm with a relatively narrow angle, this result is because the corresponding lines are located towards the left in the reference image and inversely towards the right in the shift image. The matching results for the corresponding lines are thus affected by the distortion in opposite directions, highlighting that the distance between the corresponding lines increases and decreases in opposite directions. Contrarily, in a pair of stereo images acquired at an 80 mm working distance with a relatively wide-angle, the corresponding lines of both the reference and the shift images are located towards the left—i.e., in the same region. The distance between the corresponding lines thus gradually increases.

**Figure 5:**
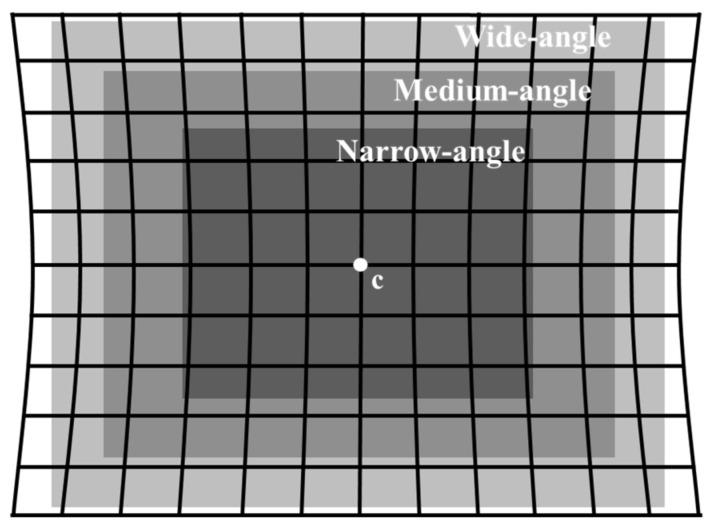
The distortion ratio according to the different angles of the image.

From the results of Figure 4, we confirm that the distortion ratio of the vertical line pattern image is different according to each working distance between the mobile camera lens and the image. As can be seen in Figure 5, the range of view depends on the working distance—i.e., the higher the working distance, the wider the image we can acquire. The larger the range at which the image is taken—that is, the wider the angle of view of the image—the stronger the distortion. Therefore, we calculated the maximum distortion ratio of the images with different angles according to changes in the working distance. We adopted the maximum value of the distance between the corresponding line at each working distance as the maximum distortion ratio. Figure 5 shows that the degree of distortion in the image increases according to the increase in the angle of the image.

**Figure 6:**
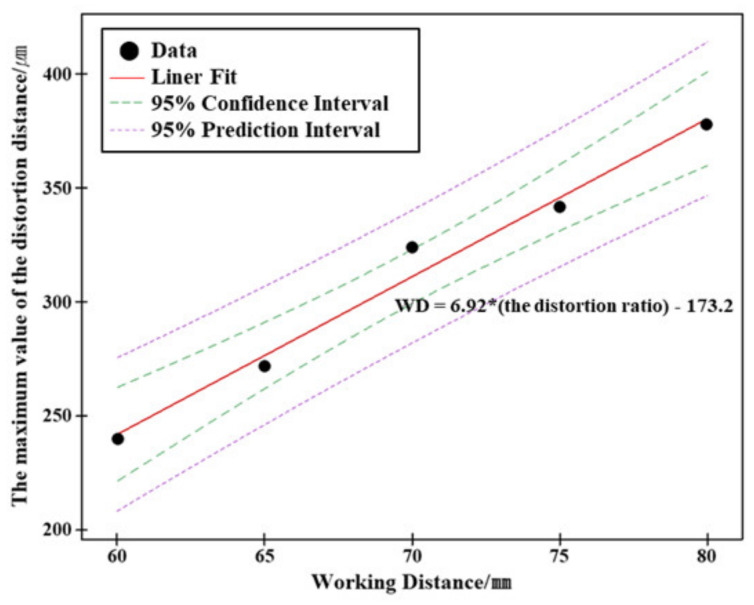
Regression analysis on the image angle (at five different working distances) and the distortion ratio (the maximum values of the distance between the corresponding lines).

To analyze the relationship between the distortion ratio and the mobile image angle, we performed a linear regression analysis using the maximum value of the distance between the corresponding lines in the stereo images acquired at each working distance. We then obtained a simple linear regression model with the equation: working distance $=6.92\times \left(the\;distortion\;ratio\right)-173.2$ for the image angle and distortion ratio. The obtained model fits the distortion ratio, according to the image angle, up to 98.28% (Figure 6). Additionally, we verified that a simple linear regression model could explain the relationship between the image angle and distortion ratio; in other words, the results of the regression analysis are meaningful (p=0.001).

**Figure 7:**
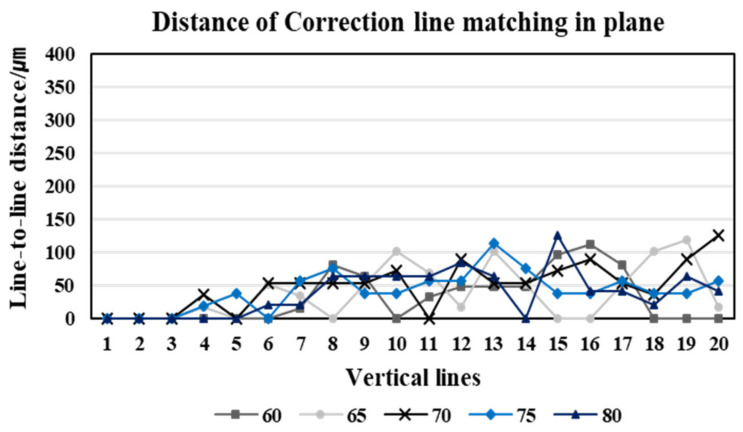
The stereo matching results of the corrected stereo images in the plane.

Next, we corrected the distorted stereo images for the vertical line pattern using the DCM and matched corresponding lines. In the case of images acquired using the same camera lens, we corrected the images for distortion by applying an identical DCM, since the images from different angles resulted in identical image distortion. In the graph provided in Figure 7, the corresponding distance is reduced to nearly zero in the matching results of the stereo images and hence confirmed that the image correction was appropriately applied. After matching the position of the first corresponding line, we proceeded to match the following lines. Thus, if a distance error occurs between the first corresponding lines, the following lines are also affected. Thus, the 2nd to 20th matching distance, as seen in Figure 7, has a larger error than the actual distance error.

Table 1 shows the correction rate as the distance variation in the corresponding lines before and after the distortion correction. The correction rate is calculated by the following equation, where $d_{di}$ denotes the distance between the corresponding lines of the distorted image and $d_{ci}$ denotes the distance between the corresponding lines of the corrected image.

(5) $$Average\;Correction\;rate\left(\%\right)=\frac{1}{20}\sum_{i=1}^{20}\frac{\left|d_{di}\right|-d_{ci}}{\left|d_{di}\right|}\times 100.$$

For close-up images captured using a mobile phone, a fine distortion was observed. Therefore, after distortion correction fine error values can have a significant impact on the correction rate. The maximum error of the correction was approximately 0.1 mm, which is very fine. This error has little visual impact on the image captured by the mobile phone.

### 3.3. Comparing before-and-after Distortion Correction for Mobile Stereo Pattern Images Containing Depth

**Figure 8:**
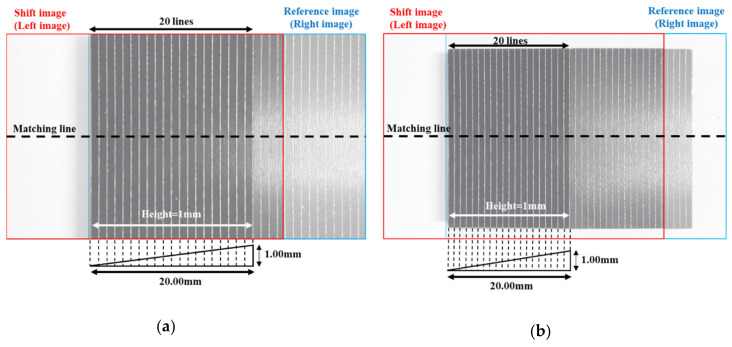
The combining image of the mobile stereo images with depth (i.e., combining the reference (in blue) and shift (in red) images). (**a**) Stereo images are acquired by the working distance 60 mm, (**b**) stereo images are acquired by the working distance 80 mm.

For the mobile stereo images of the standard scalar bar with depth [7], we compared and analyzed the distortion correction before and after by matching the corresponding lines given in the left and right images. Figure 8 shows stereo images of the standard scalar bar. The matching 20 lines differ in height and have a total height difference of 1 mm, with an interval of 1 mm between each line. Moreover, we acquired stereo images with a stereo baseline of 10 mm at five different working distances.

**Figure 9:**
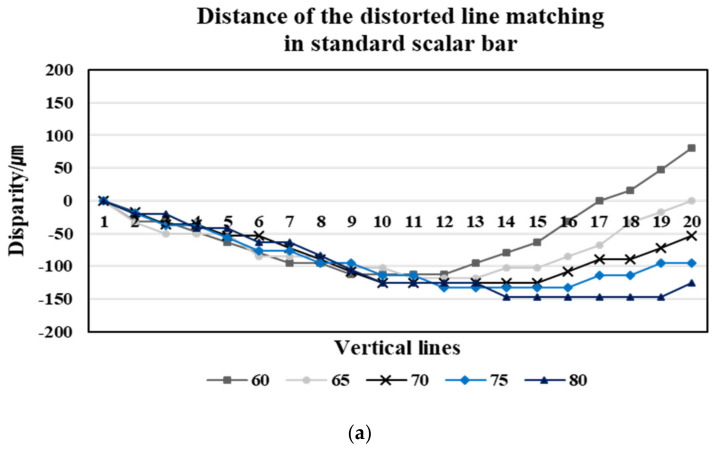

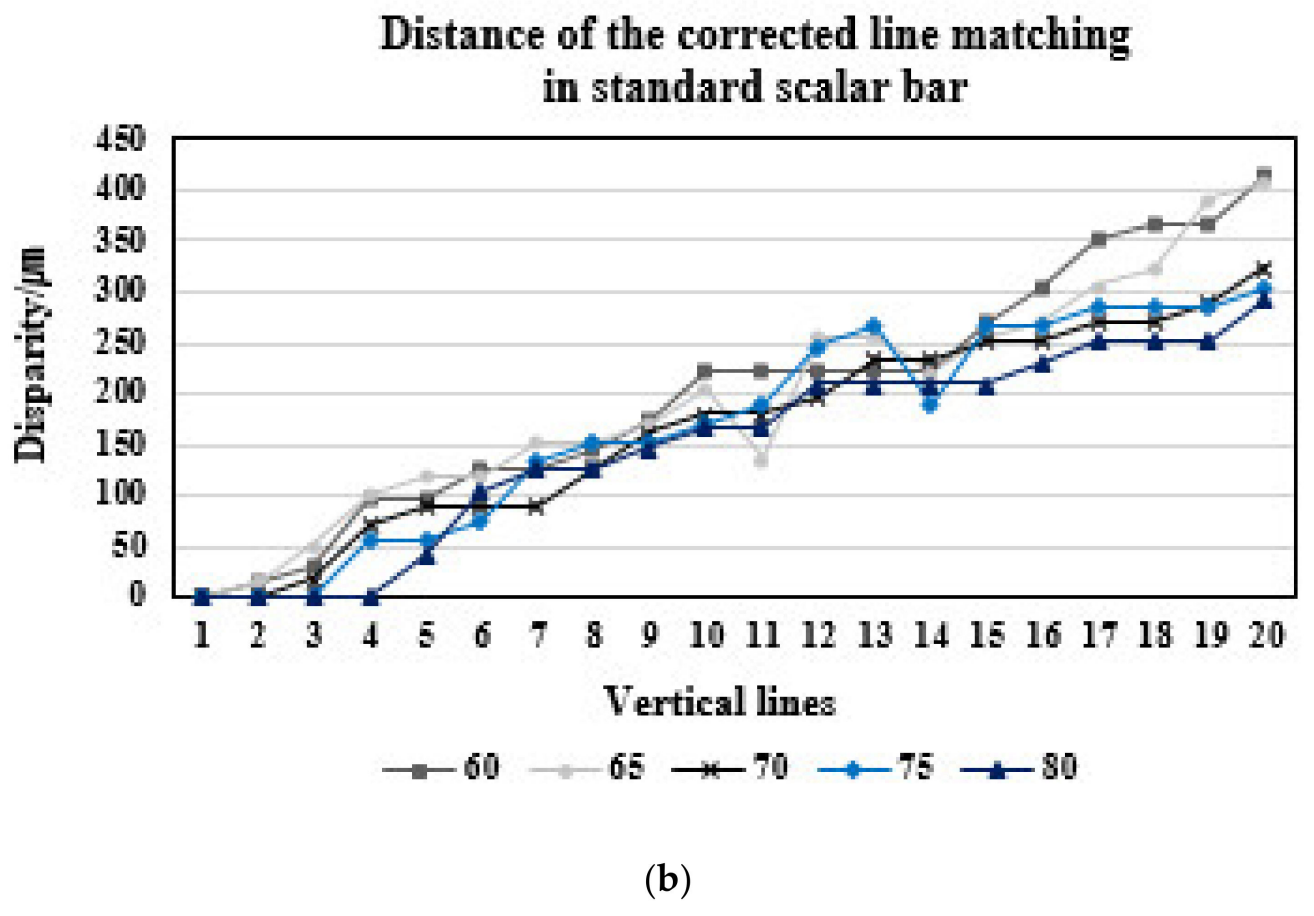
The matching result of the stereo image for a vertical line pattern, including the depth information. (**a**) The matching result of the distorted stereo image, (**b**) the matching result of the corrected stereo image.

We calculated the distance = $x_{Li}-x_{Ri}$ in the stereo images of the standard scalar bar, which is the same process of stereo images in the plane. Here, the distance denotes the disparity that represents the depth of 3D information. Disparity d, depth z, baseline b, and focal length f are related by the following relationship: d=bf/z. The larger the distance between the stereo images (baseline b) and the smaller the working distance of the images (depth z), the greater the value of d. Based on this relation, we compared before and after the correction of stereo images of the standard scalar bar using the matching results of the corresponding lines. If the image does not have distortion, the disparity increases as we move from the first corresponding line towards the 20th corresponding line. However, when looking at the matching results of the distorted stereo images in Figure 9a, the corresponding line of the right image positioned toward the left direction is significantly affected by the distortion, resulting in $x_{Li}<x_{Ri}$. Therefore, at first it can be seen that the difference in the calculated distance increases to a negative value; after we pass through the corresponding lines positioned in the central region, where a small distortion effect is observed, it then turns positive. These result patterns are the same as the matching results of the stereo pattern images in the plane. Therefore, the effect of distortion on the mobile image is strong regardless of the information of depth.

The graph confirms that as a result of distortion correction, the stereo matching results—i.e., disparity—match the stereo vision principle (Figure 9b). Furthermore, depending on the relationship with the working distance at which the images are taken, we observe that the shorter the working distance z, the greater the disparity value d. Comparing the vertical line pattern images that include depth information before and after correction, we observe that the geometric changes in the matched lines are caused by distortion, thus that causes problems while extracting accurate 3D information from the mobile image. We can detect the depth difference of 50 µm for each line, as well as total depth differences of 1 mm through the corrected stereo image. In other words, through Figure 9b, which is disparity graph acquired through the matching of the corrected standard scalar stereo image, we can confirm different disparity values according to the different depths for each line. As a result, we can correct the distorted stereo images, including the fine depth information, which enable us to extract 3D information using the corrected close-up mobile image.

### 3.4. The Correcting Distortion Result of the Mobile Skin Image

**Figure 10:**
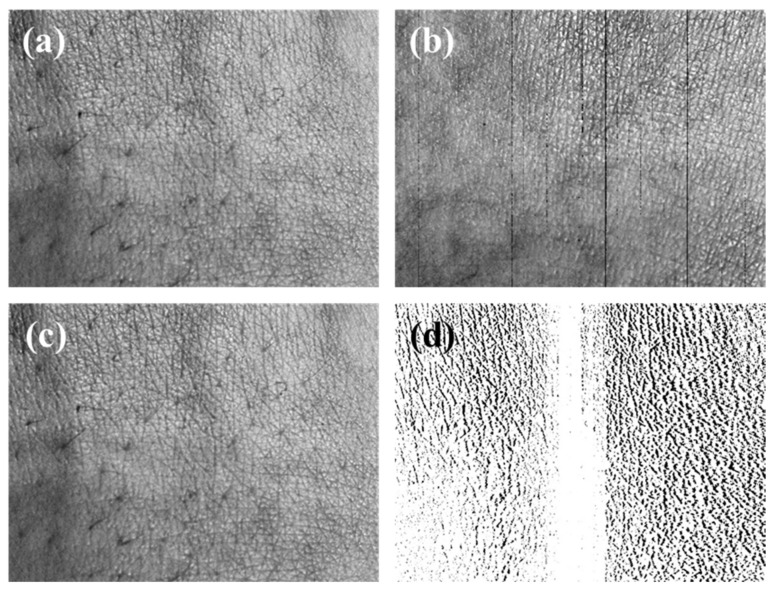
The distortion correction results of the mobile skin image acquiring the working distance of 60 mm. (**a**) The distorted original skin image, (**b**) the corrected skin image, (**c**) the interpolation skin image, (**d**) the subtraction image from the distorted image and the interpolation image.

**Figure 11:**
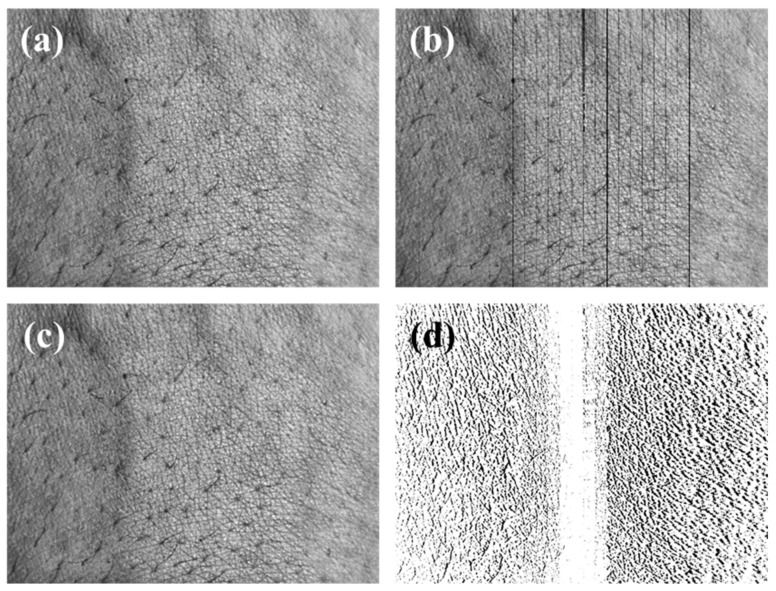
The distortion correction results of the mobile skin image acquiring the working distance of 80 mm. (**a**) The distorted original skin image, (**b**) the corrected skin image, (**c**) the interpolation skin image, (**d**) the subtraction image from the distorted image and the interpolation image.

**Figure 12:**
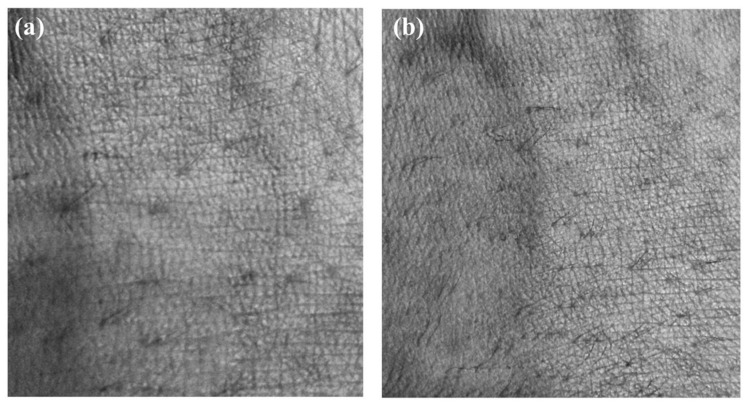
The overlap image of the reference and shift image obtained when the baseline was 10 mm. (**a**) Overlap image acquired by the working distance 60 mm, (**b**) overlap image acquired by the working distance 80 mm.

As a result, we corrected the distortion of the mobile skin image using the DCM, as verified through the previous results, and performed the interpolation of the hole region. Since it is difficult to check fine distortion correction with the naked eye, we confirmed the degree of correction using the subtraction image before and after the correction. The subtraction image is what subtracts the corrected image from the distorted image. We also corrected the skin images of different angles. A comparison shows that the interpolated image (c) is similar to the original image (a). As seen in the subtraction image (d), the distortion occurs on both sides of the image, and this corrected difference influences the accuracy of the three-dimensional analysis of the skin image. Figure 10 and Figure 11, which were acquired at a working distance of 60 and 80 mm, respectively, show the correction process of the mobile skin image. The process can correct the distortion of the mobile skin images that contain complex and detailed information by employing the proposed DCM. Furthermore, the corrected image enables a more accurate and precise skin condition analysis. Figure 12 shows the overlap images of the corrected stereo skin image. After the correction distortion, the difference in position of the skin surface features shown in Figure 12 can be reliable data.

## 4. Discussion

In general, people are interested in maintaining their young and healthy skin. However, it is difficult to analyze and manage their skin condition in practice. The reliability and precision of the data acquired by means of mobile cameras were low and hence were less useful for application to the field of skin analysis. With recent advances in mobile phones, we can acquire skin-related data to analyze skin conditions. However, the geometric changes in the skin’s surface structure are affected by lens distortion in the mobile images. This problem reduces the accuracy of three-dimensional information analysis, such as the depth of wrinkles and the roughness of the skin surface. However, existing distortion correction studies have dealt with barrel distortion that appears strongly across very wide areas of images containing real-world information. Hence, the previously proposed methods were not suitable for the fine distortion correction of mobile images. Additionally, existing distortion correction algorithms calculate the distortion coefficients based on the most severe distortion region under the assumption that lens distortion occurs symmetrically in the left and right regions. However, while checking the mobile images used in this study, we confirmed that the degree of distortion on the left and right sides of the image differed depending on the center of the image.

Therefore, in this study we propose an adaptive distortion correction method using a division model to correct the fine distortion appearing in the pixels of the mobile image by pixels. Moreover, we generated a DCM capable of pixel-by-pixel distortion correction and performed the distortion correction of mobile images. We used a single pattern image, stereo pattern images without depth, stereo pattern images with depth information, and finally images of skin surface to verify the performance of the DCM. By comparing the results of stereo images before and after distortion correction, we obtained a corrected image that was similar to the undistorted ideal image.

Additionally, we analyzed the relationship between the mobile image angle and distortion ratio by acquiring mobile images at five different working distances. It was generally observed that the wider the image angle, the larger the distortion of the outer region of the image. This same phenomenon occurred in the acquired mobile image, and we presented a simple regression model that showed the relation of each image angle and distortion ratio by performing a linear regression analysis. Next, we performed stereo matching for stereo images of the standard scalar bar containing depth information. Through the result of the corrected stereo image matching, we confirmed the feasibility of the three-dimensional skin analysis by perceiving the fine depth of the object in the mobile image. In the corrected result using the DCM, we acquired the corrected image containing the hole region in the process of relocating the pixels. The hole region degrades the image quality. Thus, we used nearest neighborhood interpolation, which can express the continuous skin surface structure well, to produce a high-quality corrected skin image. This process improves the image quickly and is easy to use for real-time analysis. Actually, the image correction performance time using the generated DCM was about two seconds and could be processed quickly.

In this study, we propose a method to correct the fine distortion that appears in close-up mobile images, rarely covered in existing studies. The acquired close-up mobile image displays fine pincushion distortion, making it difficult to extract features from biometric data at the µm level. The results of our study can be used to increase the precision of the analysis of the acquired mobile biometric data and expand the utility range of mobile phones in the field of health. In future studies, a new system can be developed for the evaluation and management of skin aging or various skin diseases through the 3D analysis of the skin using corrected mobile skin images.

## 5. Conclusions

Our study proposes a pincushion distortion correction method that adjusts geometric changes in the skin surface structure as a pre-processing step to skin analysis for extraction 3D information of skin surface, such as surface roughness and depth of wrinkles, using stereo images of the skin surface captured using a mobile phone. We tested the lens distortion correction by acquiring two types of images; one was a vertical line pattern image in-plane, and the other was a vertical line pattern image containing depth information. We analyzed each stereo image, compared the before-and-after correction distortion, and verified the performance of our proposed method. Additionally, we analyzed the relationship between the mobile image angle and the distortion ratio. By matching the stereo images, including the depth information, we confirmed that it is possible to perceive fine depth information at the µm level in the corrected mobile image. Prior studies on distortion correction dealt with significant distortions visible to the naked eye. The correction results showed considerable changes before and after correction. However, our study dealt with fine distortions which are difficult to identify with the naked eye. Additionally, prior studies on skin surface analysis performed research by selecting the region of the skin with a small distortion effect on the skin image, thus having a limitation in the observation region. To overcome this limitation, the proposed fine distortion correction method enables us to use the entire region of the mobile image and observe regions about eight times wider than the existing skin observation region [11]. The proposed correction distortion method, DCM, can be utilized and applied for the correction of various kinds of camera lens distortion. The results of our study can be used to expand the utilization of mobile cameras in other fields as well as extract reliable and accurate data through mobile images.

## Figures and Tables

**Table 1:** The maximum error of the corresponding lines in the corrected stereo images and the correction rate according to the working distance.

Working Distance (mm)	Average Correction Rate (%)	Maximum of Error Distance before Correction (mm)	Maximum of Error Distance after Correction (mm)
60	84.6%	0.24	0.11
65	82.7%	0.27	0.12
70	80.7%	0.32	0.13
75	81.7%	0.34	0.11
80	86.3%	0.38	0.13

## References

[1] Rew J., Choi Y.-H., Kim H., Hwang E. (2019). Skin Aging Estimation Scheme Based on Lifestyle and Dermoscopy Image Analysis. Appl. Sci..

[2] Hong G., Lee O. (2019). Three-Dimensional Reconstruction of Skin Disease Using Multi-View Mobile Images. Ski. Res. Technol..

[3] Zhang J., Zhang Y., Chen B. (2017). Out-of-Focus Projector Calibration Method with Distortion Correction on the Projection Plane in the Structured Light Three-Dimensional Measurement System. Sensors.

[4] Tan L., Wang Y., Yu H., Zhu J. (2017). Automatic Camera Calibration Using Active Displays of a Virtual Pattern. Sensors.

[5] Catherwood T.E., Winder J.A., McIntosh S.A., Winder R.J. (2014). 3D Stereophotogrammetry: Post-Processing and Surface Integration. Imaging Sci. J..

[6] Ko M., Kim N., Kim K. (2019). Accurate Depth Estimation of Skin Surface Using a Light-Field Camera Toward Dynamic Haptic Palpation. Ski. Res. Technol..

[7] Lee O., Lee G., Oh J., Kim M., Oh C. (2010). An Optimized In Vivo Multiple-Baseline Stereo Imaging System for Skin Wrinkles. Opt. Commun..

[8] Lee O., Lee G., Kim M., Kim S.-K., Baek Y., Oh C. (2013). Multimodal Evaluation of Xenograft Tumors in Mice with an In-Vivo Stereo Imaging System and Small-Animal PET/CT. Melanoma Res..

[9] Freire-Obregón D., Narducci F., Barra S., Castrillón-Santana M. (2019). Deep Learning for Source Camera Identification on Mobile Devices. Pattern Recognit. Lett..

[10] Xu J., Ding H., Yu Z., Zhang Z., Liu W., Chen X. (2020). Joint Space-time Coding and Power Domain Non-orthogonal Multiple Access for Future Wireless System. KSII Trans. Internet Inf. Syst..

[11] Moon C.-I., Lee O. (2018). Age-Dependent Skin Texture Analysis and Evaluation Using Mobile Camera Image. Ski. Res. Technol..

[12] Shih Y., Lai W.-S., Liang C.-K. (2019). Distortion-Free Wide-Angle Portraits on Camera Phones. ACM Trans. Graph..

[13] Wu F., Wei H., Wang X. (2017). Correction of Image Radial Distortion Based on Division Model. Opt. Eng..

[14] Layek A., Chung T., Huh E.-N. (2019). Remote Distance Measurement from a Single Image by Automatic Detection and Perspective Correction. KSII Trans. Internet Inf. Syst..

[15] Wang X., Liu J., Zhou Q. (2016). Real-Time Multi-Target Localization from Unmanned Aerial Vehicles. Sensors.

[16] Benligiray B., Topal C. Blind Rectification of Radial Distortion by Line Straightness. Proceedings of the 2016 24th European Signal Processing Conference (EUSIPCO).

[17] Wu R.Q., Liu J., Chen W., Gu Q.S. (2019). Adaptive Wide-Lens Distortion Correction Based on Piecewise Polynomial Optimization. Procedia Comput. Sci..

[18] Zheng B., Dong Y., Mullany B., Morse E., Davies A. An Optical Positioning Sensor by Combining Optical Projection and a Virtual Camera Model. Proceedings of the Remote Sensing System Engineering V.

[19] Hartley R., Zisserman A. (2003). Multiple View Geometry in Computer Vision.

[20] Fitzgibbon A.W. Simultaneous Linear Estimation of Multiple View Geometry and Lens Distortion. Proceedings of the 2001 IEEE Computer Society Conference on Computer Vision and Pattern Recognition, CVPR 2001.

[21] Lee M., Kim H., Paik J. (2019). Correction of Barrel Distortion in Fisheye Lens Images Using Image-Based Estimation of Distortion Parameters. IEEE Access.

[22] Qiao N. Distortion correction of photoelectric image acquired by CCD camera. Proceedings of the Sixth International Conference on Optical and Photonic Engineering (icOPEN 2018).

[23] Park J., Byun S.-C., Lee B.-U. (2009). Lens Distortion Correction Using Ideal Image Coordinates. IEEE Trans. Consum. Electron..

